# Long-Term Follow-Up of Cushing’s Disease: A Case Report

**DOI:** 10.4274/Jcrpe.993

**Published:** 2013-09-18

**Authors:** Veysel Nijat Baş, Sebahat Yılmaz Ağladıoğlu, Aşan Önder, Pınar Özışık, Havva Nur Peltek Kendirci, Semra Çetinkaya, Zehra Aycan

**Affiliations:** 1 Dr. Sami Ulus Women Health, Children’s Education and Research Hospital, Clinics of Pediatric Endocrinology, Ankara, Turkey; 2 Social Security Institution Children’s Hospital, Clinics of Pediatric Neurosurgery, Ankara, Turkey; 3 Yıldırım Beyazıt University, Clinics of Pediatric Endocrinology, Ankara, Turkey

**Keywords:** obesity, growth rate, Cushing’s disease

## Abstract

Cushing’s disease is a condition in which hypercortisolism develops due to excessive hypophyseal adrenocorticotropic hormone production. It is rare in childhood. In this paper, we report the case of a 10-year-old male patient with hypophyseal microadenoma-related Cushing’s disease who presented with obesity and was found to show poor height growth at follow-up. The diagnosis was confirmed with inferior petrosal sinus sampling, and the adenoma was successfully removed by transsphenoidal surgery. While adrenal axis suppression continued for approximately 1 year, clinical improvement was clearly observed after the third month following surgery. The findings in this patient demonstrate that decreased growth rate despite rapid weight gain in children can be early sign of Cushing’s disease and emphasize the importance of monitoring of growth in obese children.

**Conflict of interest:**None declared.

## INTRODUCTION

Cushing’s syndrome is the clinical entity resulting from abnormally high levels of cortisol or other glucocorticosteroids. Cushing’s disease is defined as hypercortisolism due to excessive adrenocorticotrophic hormone (ACTH) production. The disorder caused by ACTH of non-pituitary origin is known as ectopic ACTH syndrome (1). Cushing’s syndrome commonly occurs due to exogenous systemic glucocorticoids, administered for treatment (2). While the most common cause of Cushing’s syndrome is Cushing’s disease in adults and in children over the age of 7 years, adrenal tumors and particularly adrenal carcinomas are the most frequent cause of the syndrome in children under the age of 7 years (3,4,5,6). Although Cushing’s disease is rarely observed in children, it is important to consider the possibility of this entity for the establishment of early diagnosis as typical clinical findings will become manifest in time (5,6). The purpose of this article is to report a long-term follow-up of a case diagnosed as Cushing’s disease after presenting to the clinic with a complaint of excess weight and observation of decreased growth rate during the patient’s follow-up.

## CASE REPORTS

The 10-year-old male patient presented to the clinic with a complaint of rapid weight gain over the past year. There was a history of type 2 diabetes in the family. The patient was prepubertal. His blood pressure was 100/60 mm/Hg. Height was 134.6 cm [-0.4 standard deviation (SD)] and body weight was 48.1 kg (+1.9 SD). Body mass index (BMI) was 26.3 (+2.3 SD). Physical examination revealed acanthosis nigricans on the neck (Figure 1a). The patient’s biochemical parameters were within normal range, and his morning and evening cortisol levels were 16.9 mcg/dL and 3.2 mcg/dL, respectively. An oral glucose tolerance test was performed, and the results were consistent with hyperinsulinism and insulin resistance. The patient was started on metformin treatment. On the 6th month of follow-up, the patient had a height of 135.6 cm (-0.7 SD). Body weight was 52.6 kg, and body mass index was 28.6 (+2.6 SD). Noting the inadequacy of a 1-cm increase in height despite the 4-kg increase in body weight, serum cortisol levels (day-night) were repeated and the results revealed a disrupted cortisol rhythm with a morning serum cortisol level of 23 mcg/dL and an evening cortisol level of 10 mcg/dL, along with elevated 24-hour urinary free cortisol levels assessed over three days (652 mcg/day, 884 mcg/day, and 674 mcg/day, respectively). Cushing’s syndrome was suspected due to lack of suppression in the low-dose dexamethasone suppression test (ACTH: 16.2 pg/mL, cortisol: 16 mcg/dL), and the diagnosis of Cushing’s disease was confirmed by the demonstration of suppression in the high-dose dexamethasone suppression test (ACTH: 5.9 pg/mL, cortisol: 1.6 mcg/dL). The evaluation of the case in terms of the systemic effects of hypercortisolism showed normal bone mineral density and no hypertensive eye-ground changes. The 24-hour blood pressure readings were consistent with systolic and diastolic hypertension. The hypophyseal magnetic resonance imaging results were reported to be normal, and the results of inferior petrosal sinus sampling were consistent with rightward lateralized microadenoma (Table 1). The pathology results of the microadenoma that was removed by transsphenoidal surgery were evaluated to be consistent with ACTH-secreting adenoma. Hydrocortisone treatment was continued(7 mg/m2/day) due to the ongoing suppression of the adrenal axis following surgery. The growth rate of the patient increased up to 6 cm/year over the duration of the one-year follow-up, and his BMI decreased from +2.6 to +0.6 (Figure 1b). On the 12th month of treatment, the peak serum cortisol response of 28 mcg/dL in a low-dose ACTH stimulation test was determined to be sufficient, and the hydrocortisone treatment that was initially started at physiological dosage was gradually stopped. The physical examination findings and basal cortisol measurement values in the follow-up period are presented in Table 2.

## DISCUSSION

Cushing’s disease is rarely observed in children and its clinical symptoms differ from those in adults. Also, the appearance of typical cushingoid symptoms may take a long time to appear, therefore, the typical symptoms of Cushing’s disease may not be observed in childhood. Abnormal growth rate is expected in obese children. Therefore, cessation of height growth should be considered as one of the earliest signs of Cushing’s disease (1,4,5,6).Our patient presented with complaints of excessive weight gain, and Cushing’s disease was diagnosed at follow-up, following the observation of decreased growth rate. The findings in this patient demonstrate the importance of monitoring growth in obese children.

Diagnosis of Cushing’s disease is based on demonstration of hypercortisolemia by biochemical laboratory tests, which are requested upon suspicious clinical findings. The most sensitive indicator of hypercortisolemia in children is the absence of diurnal rhythm. The classic characteristic of this disease is the lack of suppression in low-dose dexamethasone tests but its presence in high-dose dexamethasone tests (1,3,4). In our case, the diurnal rhythm was initially present, however, it disappeared during the follow-up period, at which time, elevated serum cortisol levels were also noted.

Pituitary imaging should be performed in patients suspected of having Cushing’s disease, suggested by the biochemical data. However, if hypophyseal lesions cannot be observed in the imaging results, bilateral inferior petrosal sinus sampling should be considered, although it poses a risk in childhood (1,3,6,7). In the present case, the potential microadenoma could not be observed in the imaging results and its localization was achieved by bilateral inferior petrosal sinus sampling.

Transsphenoidal hypophysectomy is the first-line treatment option in patients with Cushing’s disease (5). The signs and symptoms of hypercortisolism start improving within a few weeks following surgery. Improvement in the hypothalamic-pituitary-adrenal axis and significant catch-up growth are observed between 6 to 12 months following the intervention. Corticosteroid replacement therapy is necessary for adrenal insufficiency until normal adrenal function is restored. Although basal cortisol levels are normal, there is a possibility of insufficient stress response in these patients. Careful monitoring for the first few months is necessary, until improvement occurs in the functioning of the hypothalamic-pituitary-adrenal axis (1,7,8). As the first-line treatment option, surgery was successfully performed in the present case without any complications. Replacement therapy with hydrocortisone was continued until improvement in the hypothalamic-pituitary-adrenal axis was achieved at the end of the 12th month after surgery.

In conclusion, the findings in our patient demonstrate that slowing of height growth despite rapid weight gain can be an early sign of hypercortisolism in children. This finding is important for suspicion of and consequently for early diagnosis and treatment of Cushing’s disease in children.Diagnosis of Cushing’s disease is based on

## Figures and Tables

**Table 1:**
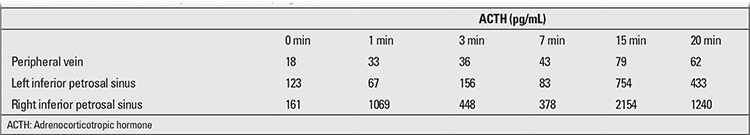
Results of bilateral inferior petrosal sinus sampling (ACTH levels)

**Table 2:**
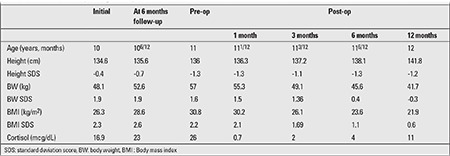
Initial and follow-up findings in the patient

**Figure 1a:**
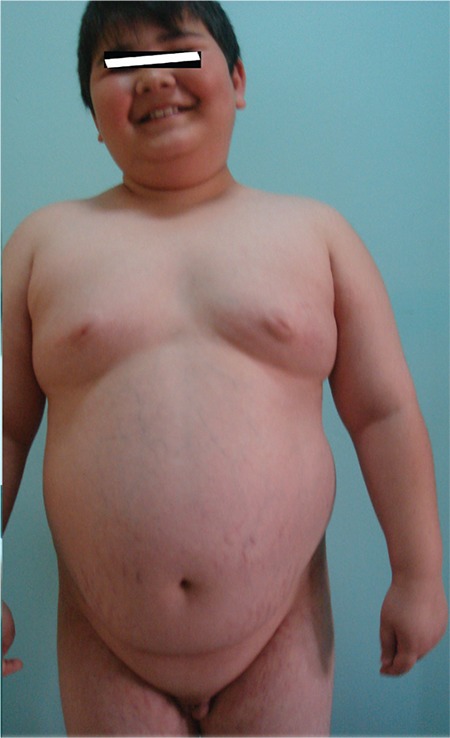
General appearance of the case before the operation

**Figure 1b:**
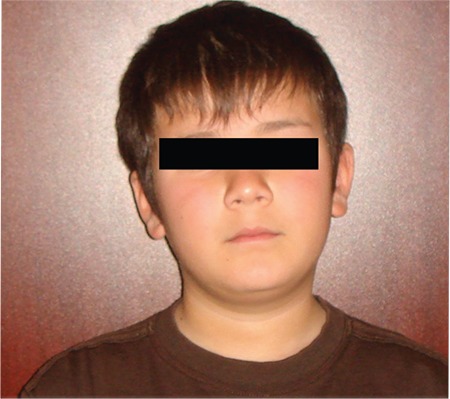
General appearance of the case after the operation
